# In silico analysis and modeling of putative T cell epitopes for vaccine design of Toscana virus

**DOI:** 10.1007/s13205-014-0247-4

**Published:** 2014-08-26

**Authors:** Amisha Jain, Pranav Tripathi, Aniket Shrotriya, Ritu Chaudhary, Ajeet Singh

**Affiliations:** 1 Department of Biotechnology, G B Pant Engineering College, Pauri Garhwal, Uttarakhand India; 2 Department of Biotechnology, Delhi Technological University, Delhi, India

**Keywords:** Toscana virus, ProPred, Structural modeling, Docking, Molecular dynamics, Vaccine design

## Abstract

The sandfly fever Toscana virus is an important etiological agent known to cause human neurological infections in endemic Mediterranean countries during summer season. In the present study, prediction and modeling of T cell epitopes of Toscana virus (TOSV) antigenic proteins followed by the binding simulation studies of predicted highest binding scorers with their corresponding MHC class II alleles were done. Immunoinformatics was applied in computational vaccinology to analyze the viral proteins which generate possible outcomes to elicit vaccine for TOSV. Here, immunoinformatic tool ProPred was used to predict the promiscuous MHC class II epitopes of viral antigenic proteins. The molecular modeling of the selected epitopes as well as MHC alleles was done at CPH model 3.2 server. Molecular dynamics (MD) simulation studies were performed through the NAMD graphical user interface embedded in visual molecular dynamics. The epitope/peptide VKMMIVLNL of viral nucleoprotein as well as VMILGLLSS of viral glycoprotein has shown the highest binding score with the same DRB1*1104 MHC II allele. These two predicted peptides are highly potential to induce T cell-mediated immune response and are expected to be useful in designing epitope-based vaccines after further testing. The results signify that the nucleoprotein, glycoprotein or the combination of both could be useful for future development of a vaccine controlling the spread of this emerging virus that could pose a new threat for humans.

## Introduction

Toscana virus (TOSV) is an arbovirus (arthropod-borne virus), which belongs to the family *Bunyaviridae* and genus *Phlebovirus* (Bishop et al. 1990; Elliott et al. 1991). There are at least 45 members of the Phlebovirus genus, which are mostly transmitted to vertebrates by phlebotomine sandflies. Its genome is comprised of an enveloped negative-strand RNA that consists of three segments, designated as large (L), medium (M) and small (S), which encodes the viral RNA polymerase, the envelope glycoprotein and the nucleoprotein, respectively (Olal et al. 2014). The segment L is the largest about 6,400 nucleotides, M is about 4,200 nucleotides and S about 1,900 nucleotides in length. TOSV is among the three most prevalent viruses associated with meningitis during the warm seasons and, therefore, must be considered as an emerging pathogen. Currently, there is no effective antiviral treatment or vaccine for the infection caused by TOSV; the best prevention will likely to come by development of effective and safe vaccine.

*Naples* and *Sicilian* serocomplexes are the two main serocomplexes of sandfly fever viruses associated with human diseases. Sandfly fever Toscana virus (SFTV) belongs to *Naples* serocomplex and is endemic in Mediterranean countries like Italy, Portugal, Spain, and Cyprus. Within the group of sandfly-transmitted *phleboviruses*, TOSV is the only virus known to cause Central Nervous System (CNS) manifestations (Braito et al. 1998; Cusi et al. 2010). Data from Italy, France and Spain indicate that it is among the three leading causes of aseptic meningitis during periods of sandfly activity (Charrel et al. 2005). The other viruses are associated with systemic febrile illness, but not CNS infections. TOSV is the most important etiologic agent of meningitis, meningoencephalitis, or encephalitis associated with fever, severe frontal headache, vomiting, myalgia, ocular pain and, nuchal rigidity (Braito et al. 1998; Dionisio et al. 2001; Nicoletti et al. 1991; Valassina et al. 1998; 2000).

Vaccination is generally considered as a cost-effective weapon for preventing life-threatening infectious diseases. Awareness of differences in epitopes recognized by T cells and B cells has enabled immunologists to begin designing vaccine candidates to maximize activation of both arms of the immune system. Major histocompatibility complex (MHC) plays an important role in intercellular recognition of foreign antigenic peptides and thus participates in the development of both humoral and cell-mediated immune responses. T cells are present on the surface of antigen-presenting cells (APCs) and they recognize antigenic fragments only when they are combined with MHC molecules exposed on the surface of all vertebrate cells (Shekhar et al. 2012; Mohabatkar and Mohammadzadegan 2007). MHC molecules are heterodimeric glycoproteins which present a highly diverse set of peptides on the surface of a cell and thus induce T cell activation, hence play a profound role in immune response regulation (Viret and Janeway 1999; Tambunan and Parikesit 2011). As they are highly polymorphic in nature, the binding region for one allele may not induce an immune response in a population with different alleles. This raises the need for the identification of promiscuous viral peptides which can bind to multiple MHC alleles (Jiao et al. 2012).

Epitope- or peptide-based vaccines offer several advantages over conventional vaccines as they are more specific, easy to produce, cost-effective, less time-consuming and also safe. Apart from these, the main advantage of epitope- or peptide-based vaccine is the ability to deliver high doses of potential immunogen at low cost (Von Hoff et al. 2005; Tang et al. 2012). The viral protein that could act as a vaccine candidate must be antigenic and responsible for pathogenicity (Cerdino-Tarraga et al. 2003; Verma et al. 2011).

## Materials and methods

### Retrieval of Toscana virus antigenic proteins

The complete sequences of viral nucleoprotein (NP) and glycoprotein (GP) were retrieved from National Center for Biotechnology Information (NCBI), having accession code, ABY19521 and AGP51529, with length 253 and 1,339 amino acids, respectively (http://www.ncbi.nlm.nih.gov/protein).

### T cell epitope prediction by ProPred

Several different prediction methods have been employed for the identification of promiscuous T cell epitopes. Identification of various T cell epitopes by more than one MHC allele and their recognition by more than one T cell clone are known as “promiscuous” epitopes. ProPred, the graphical web-based tool was used in this study to predict MHC class II binding regions in an antigenic sequence, using quantitative matrices that assist in locating promiscuous binding regions that are useful in selecting vaccine candidates (Sturniolo et al. 1999). In ProPred, the NP and GP sequences were analyzed, taken into consideration a total of 51 MHC class II alleles. Each nonamer comprises an anchor or starting residue that helps to identify those antigenic determinants in NP and GP which binds to several Human Leukocyte Antigen (HLA) class II molecules with good binding affinity. The predicted binders can be visualized either as peaks in graphical interface or as colored residues in HTML interface. Peptides displaying highest score were selected for immunoinformatic analysis.

### Modeling and validation of favored MHC II alleles and epitopes

The NP epitope (VKMMIVLNL) and GP epitope (VMILGLLSS) of TOSV were modeled at CPH model 3.2 server. The 3-D structure of DRB1*1104 MHC allele was unavailable in the Protein Data Bank (PDB) server, therefore, CPH model 3.2 server was used to acquire its three-dimensional coordinate files employing its respective structural template (1A6A.B). The structure of MHC allele was then validated using various online structural analysis and verification servers, viz., Errat, ProQ, ProSA, RAMPAGE, etc. Errat (Colovos and Yeates 1993), a protein structure verification algorithm was especially used to analyze the statistics of non-bonded interactions between different atom types. The quality of a protein model was predicted by a protein quality predictor tool, ProQ (Wallner and Elofsson 2003) on the basis of two quality measures, LG score and MaxSub. A web-based Protein Structure Analysis tool ProSA (Wiederstein and Sippl 2007) calculates an overall quality score of specific protein structure by determining the overall model quality (Z-score) and local model quality. RAMPAGE (Lovell et al. 2002) was used for Ramachandran Plot Assessment of protein model.

### Binding simulation of epitopes with the alleles

After structural modeling, rigid docking of selected peptides and alleles was accomplished with the help of Hex 8.0 (Ritchie et al. 2008) followed by semi-flexible docking using AutoDock Vina (Trott and Olson 2010) to determine the energy minimization values. In Hex, the prediction of energy minimization is based on various parameters including correlation type of shape only, 3-D FFT mode, Grid dimension of 0.6, receptor range of 180 with step size 7.5 and ligand range of 180 with step size 7.5. Semi-flexible docking was achieved using AutoDock Vina which involves choosing of macromolecule, adding polar H-bonds, computing Gasteiger, and Kollman charges for the receptor. Then, for ligand all bonds, except amide bonds, were made rotatable. The docked complexes were finally visualized and analyzed using Autodock Tools.

### Molecular dynamics simulation of epitope and HLA allele complex

Molecular dynamics simulation was done using NAMD graphical interface module (James et al. 2005) incorporated visual molecular dynamics (VMD) (Humphrey et al. 1996). Automatic PSF builder tool of VMD was used to make the PSF file and by accessing PSF and PDB files, NAMD generated the trajectory DCD file. Root Mean Square Deviation (RMSD) of the complex was completed using rmsd.tcl source file from the Tk console and finally rmsd.dat was saved and accessed in Microsoft office excel 2007.

## Results

### Prediction and analysis of MHC Class II binding peptides

The NP peptide VKMMIVLNL at position 58–66 showed maximum binding score of 62.65 with DRB1*1104 MHC II allele. The GP peptide VMILGLLSS at position 824–832 showed maximum binding score of 73.49 with the same DRB1*1104 allele. ProPred scores and the no. of MHC II alleles showing binding affinities for epitopes are shown below (Table 1).

**Table 1:** Scores generated by ProPred

Protein	Position	Peptide/epitope	ProPred	HLA alleles	No. of reactivated
			Score		MHC alleles
NP	58–66	VKMMIVLNL	62.65	DRB1*1104	51
GP	824–832	VMILGLLSS	73.49	DRB1*1104	49

### Modeling and validation of target peptides and MHC allele

**Fig. 1:**
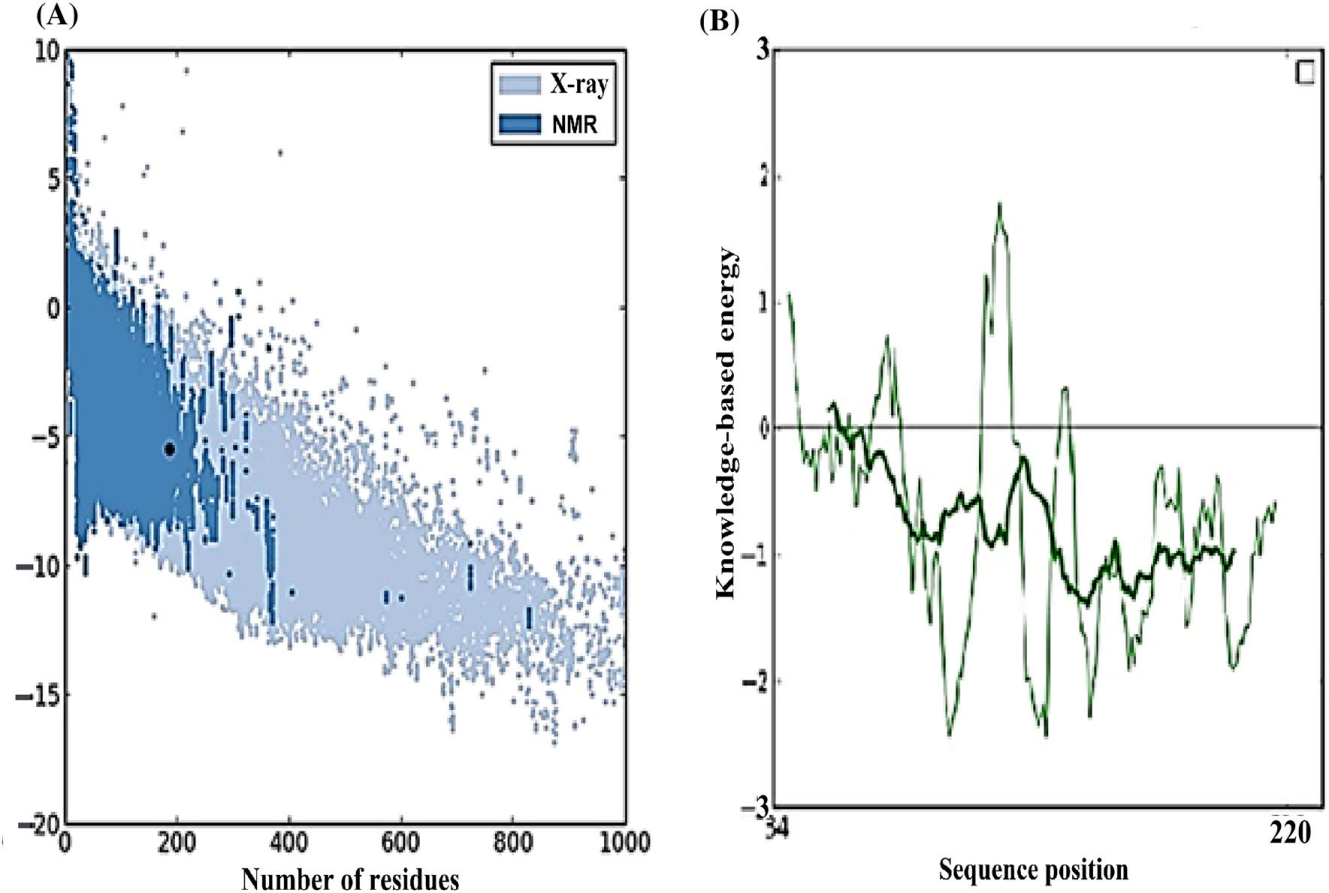
ProSA analysis. **a** Z-score plot of DRBI*1104 allele. **b** Graphical plot of residue scores of allele structure

3-D coordinate files of preferred peptides and allele were obtained through CPH model server. The structure of DRB1*1104 allele was validated using Errat, ProQ, ProSA, and RAMPAGE web-based tools. The overall quality factor of allele as determined by Errat was found to be 67.039. Quality of a model as predicted by ProQ was ‘fairly good’ with LG score of 2.096 and MaxSub of 0.131. Using ProSA, the calculated Z-score of above-mentioned allele was −5.52 (Fig. 1) and the local model quality was also good to consider. Through RAMPAGE analysis, the number of amino acid residues in favored region was 177 (95.7 %) out of 187 amino acid residues.

### Binding energy determination by Hex 8.0 and AutoDock Vina

**Fig. 2:**
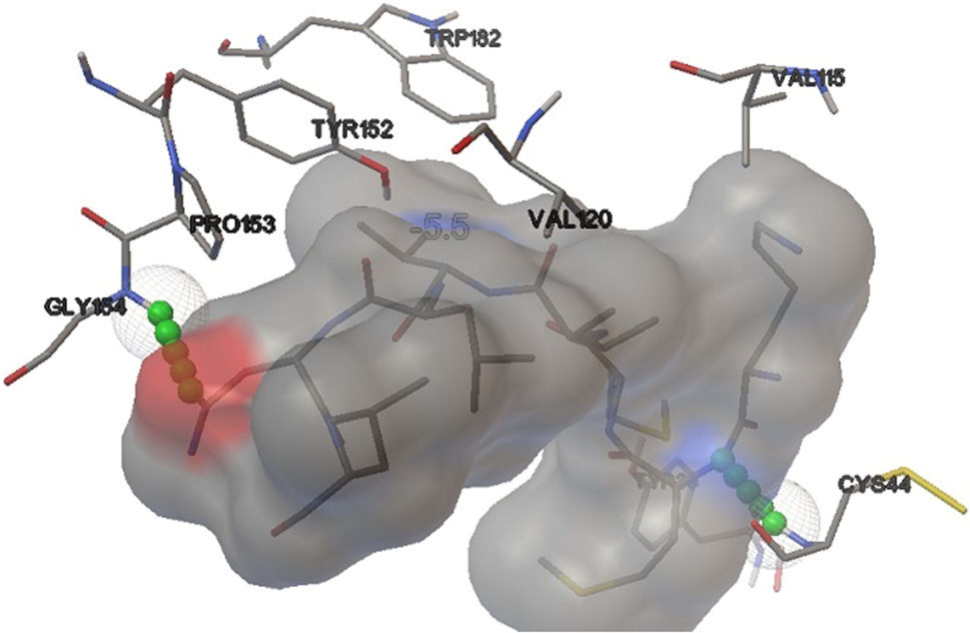
Docked NP peptide VKMMIVLNL-DRB1*1104 allele complex by Autodock Vina depicting detailed position of amino acids along with formation of 2 H-bonds with CYS44 and GLY154

**Fig. 3:**
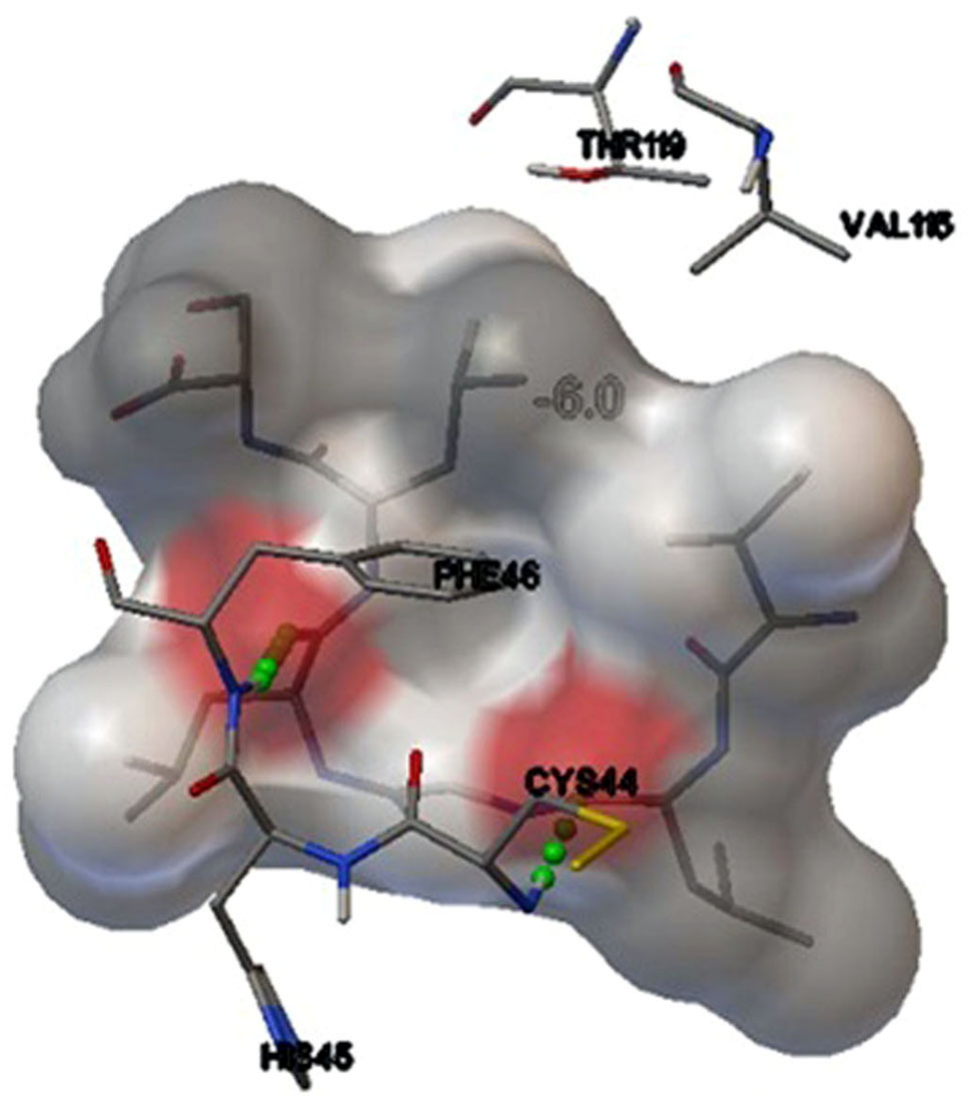
Docked GP peptide VMILGLLSS-DRB1*1104 allele complex depicting position of amino acids along with formation of 2 H-bonds with CYS44 and PHE46

Binding simulation studies of NP epitope VKMMIVLNL with DRB1*1104 allele as well as GP epitope VMILGLLSS with DRB1*1104 allele formed stable HLA–peptide complexes with the energy minimization values of −5.5 and −6.0 kcal/mol, respectively (Table 2). After docking studies, we determined the number of H-bonds present in the stable complex formed. Using AutoDock Vina, it was found that two H-bonds were present in NP-DRB1*1104 complex at positions CYS44:HN1 and GLY154:HN1 (Fig. 2), whereas two H-bonds were present in GP-DRB1*1104 complex at positions CYS44:HN1 and PHE46:HN1 (Fig. 3). While using Hex 8.0, the predicted number of H-bond was 1 in both the complexes with good E-total values. Thus, it shows maximum probability of proper binding as there is possibility of formation of H-bonds.

**Table 2:** Energy minimization results of docking studies with Hex 8.0 and Autodock Vina followed by the identification of H-bonds

Protein	Peptide/Epitope	Hex 8.0		Autodock Vina		HLA alleles
		E-total	H-bonds	Energy	H-bonds	
NP	VKMMIVLNL	−446.19	1	−5.5	2	DRB1*110
GP	VMILGLLSS	−301.04	1	−6.0	2	DRB1*1104

### Molecular dynamics simulation of peptide–allele complex through NAMD

**Fig. 4:**
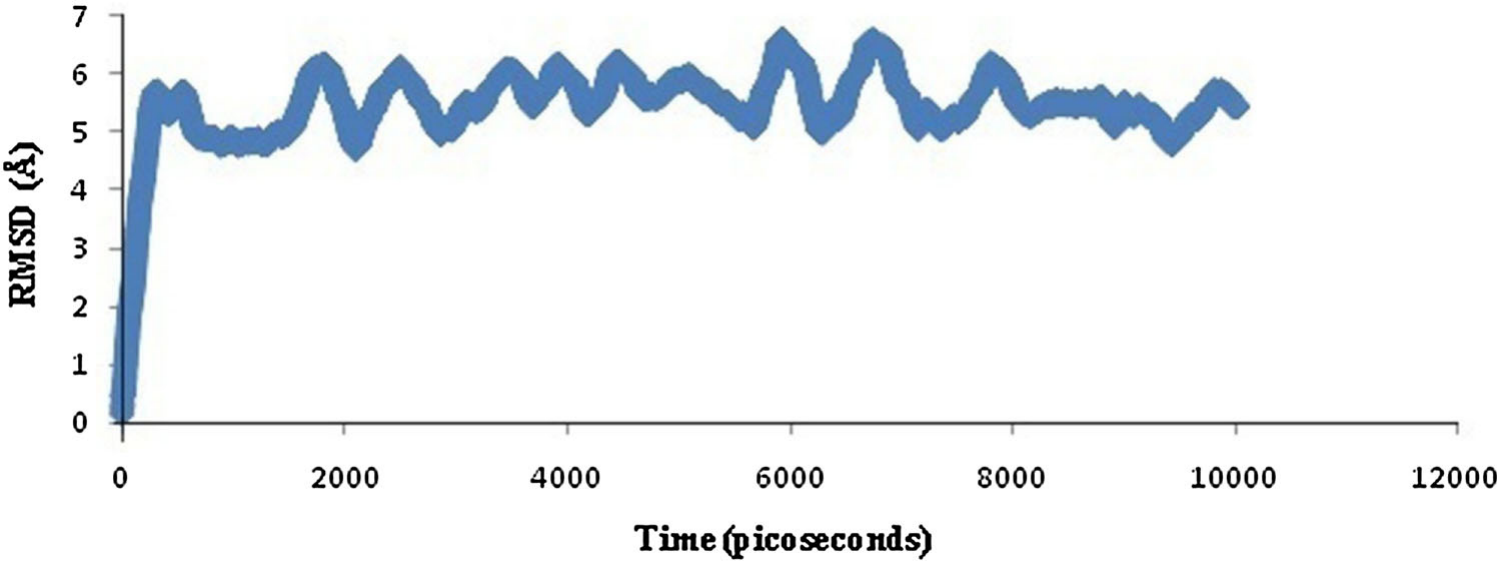
Graph displaying molecular dynamic simulation of NP–allele complex, resulted in highest peak at 6.8 Å

**Fig. 5:**
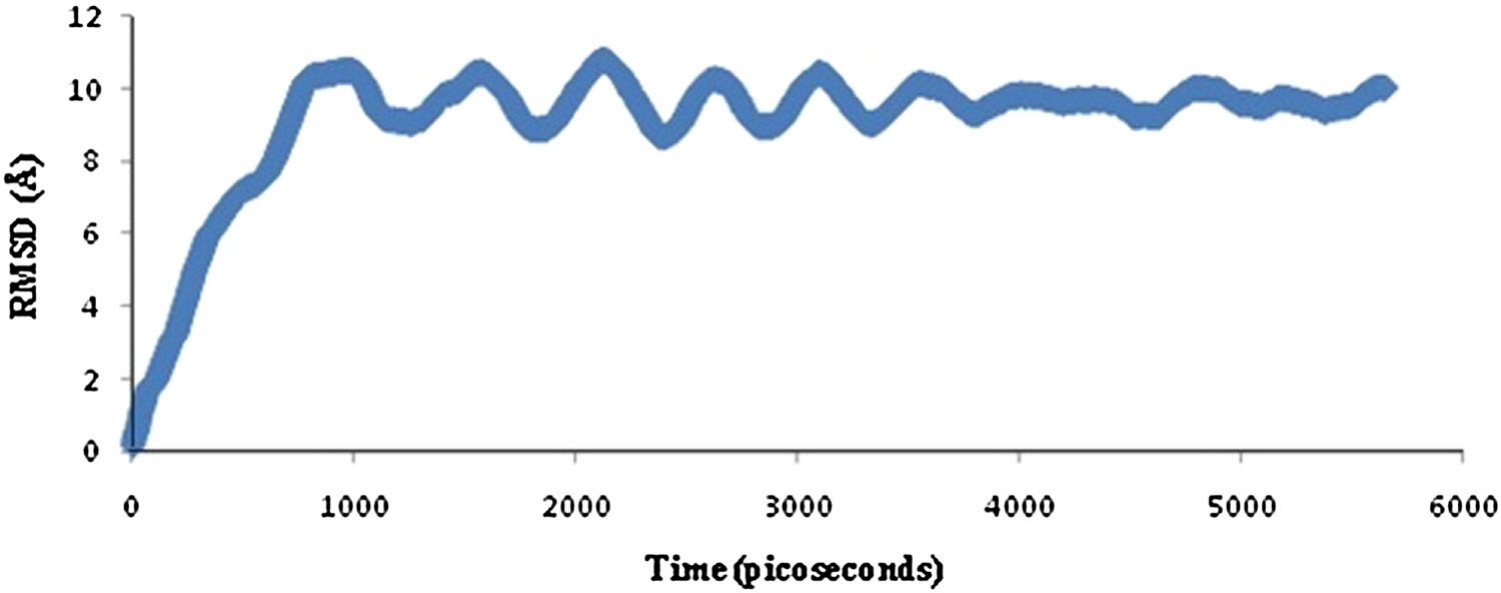
Graph displaying molecular dynamic simulation of GP–allele complex, resulted in highest peak at 11 Å

The peptide–allele complexes formed by Hex 8.0 were subjected to molecular dynamics simulation and RMSD. NP peptide VKMMIVLNL-DRB1*1104 allele complex displayed the highest peak at RMSD value of 6.8 Å (Fig. 4). On the other hand, GP peptide VMILGLLSS-DRB1*1104 allele complex resulted in highest peak at RMSD value of 11 Å (Fig. 5). As displayed by the graph, the NP–allele complex runs till 10,500 picoseconds, while the GP–allele complex runs till 6,000 picoseconds which is lesser than the time value of NP–allele complex.

## Discussion

The pathogenic activity of Toscana virus creates a challenge to develop novel vaccines. Addressing these challenges involves a time-consuming battery of in vivo and in vitro experiments. The growth of virus and isolation of peptides on a large array of experiments is also a huge and valid constraint. The current study incorporates immunoinformatics approach for reducing the time consumed in the long array of experiments to avoid hit and trial sets. Valassina et al. 2003 reported the antigenic properties of the viral proteins (NP and GP) to better understand their immunogenic role. It has been also reported that the viral NP is highly immunogenic for many members in the genus of *Phlebovirus* and is a major antigen that acts as a scaffold for packing of virus (Martin-Folgar et al. 2010; Magurano et al. 1999). A glycoprotein of length 1,339 amino acids has possibility of holding large number of epitopes. So, two proteins, one 253 amino acids long (NP) and the other 1,339 amino acids long (GP), was taken into consideration, so that T cell bound MHC II epitopes can be predicted. Only one sequence variant for each of the two proteins was studied because NP is highly conserved and according to Collao et al. 2009 geographic differences in genotype distribution relate to differences in vector distribution only. For nucleoprotein, VKMMIVLNL peptide was predicted with the highest score of 62.65 % with DRB1*1104 allele. The homology modeling of the above-mentioned allele was satisfactorily validated with an Errat score of 67.039, LG score of 2.096 by ProQ, Z-score of −5.52 by ProSA. Ramachandran plot analysis through RAMPAGE resulted in 177 (95.7 %) amino acids in favored region. The rigid docking approach was carried out successfully by Hex 8.0 on a correlation type of shape in 3-D FFT mode. The E-total value of the predicted peptide was −446.19 using Hex 8.0. Semi-flexible approach used AutoDock Vina at default parameters which resulted in binding affinity of −5.5 kcal/mol along with two H-bonds. First H-bond was detected with CYS44:HN1 and second was predicted with GLY154:HN1. The molecular dynamics simulation showed that complex formed between a peptide and allele was attaining proper stability by creating a parallelism in RMSD over a time window of 10,500 picoseconds, with the highest peak at 6.8 Å.

For glycoprotein, VMILGLLSS peptide was predicted with highest score of 73.49 % and activating same allele as by the nucleoprotein. The homology modeling of above-mentioned allele was already completed satisfactorily, so rigid docking approach was initiated by Hex 8.0 on a correlation type of shape in 3-D FFT mode. E-total value of the predicted peptide was −301.04. Autodock Vina at default parameters resulted into binding affinity of −6.0 kcal/mol along with two H-bonds, first with CYS44:HN1 and second was predicted with PHE46:HN1. The molecular dynamics simulation here also showed proper stability over a time window of 6000 picoseconds in all the frames, with the highest peak at 11 Å.

It is observed that peptides from NP as well as GP activating the same allele were providing a solid base to the prediction for vaccine design. The mentioned peptides can be either isolated or formulated for further in vitro and in vivo testings.

## Conclusion

The conclusion drawn from present study is that the two nonameric epitopes VKMMIVLNL and VMILGLLSS of NP and GP, respectively, have considerable binding with DRB1*1104 MHC class II allele and low-energy minimization values providing stability to the peptide–MHC complex. These peptide constructs may further be undergone wet lab studies for the development of targeted vaccine against Toscana virus. This would reduce the time and expenses of in vivo and in vitro experiments.
